# Oral Hygiene Excerpts

**Published:** 1906-07-15

**Authors:** N. S. Hoff


### ORAL HYGIENE EXCERPTS.

BY N. S. HOFF, D.D.S.

Read before the Detroit Dental Society, March 8, 1906.

The Committe for the evening's program has asked me to present for your consideration such quotations as have appeared during the past year in the dental journals on the subject of oral hygiene. I cannot in the brief time at my disposal make extracts of sufficient length to make the presentation comprehensive or of any considerable value in itself. I shall only present a sufficient number of citations to cover the more important papers and activities that have come under my own notice. I would say that the National Dental Association has a committee at work on this subject and through its influence some good work has been done in various parts of the country. Spasmodically it is true in most cases, but in a few instances permanent organizations have been established and are doing excellent public work for the poor and such as have no appreciation of oral cleanliness.

In the *Dental Cosmos* for March, 1905, is printed a paper on the "Care of the Teeth of the Poor," by Dr. H. L. Ambler, which in the main is an appeal for the teaching of oral prophylaxis in the public schools by the school teachers; not professional oral prophylaxis, but rather a consistent and practicable mouth toilet. He advocates the continual reference to mouth cleanliness and its advantages, as contrasted with the disadvantages of a filthy mouth which invites decay of the teeth and disease of the mouth and digestive tract. He makes it appear that such instruction is of equal if not more important than the course now being introduced, called physical education, which has for its object merely the development of a sound body, because of close attention to physiological principles of exercise, rest and proper food. He advocates coercion where persuasion is not available. He cites the fact that the City of Cleveland pays an occulist $2,000 per year to look after the eyesight of its school children. In an examination of ninety-eight school text books on physiology, he found that thirty-five contained no oral hygienic suggestions, and the other sixty-three contained an average of one and two-thirds pages per book. The City of Strasburg, Germany, has spent $8,000 for a free dental hospital for poor children. Every school child in that city must pass a dental examination on entering school and be examined twice during the year. He maintains that 10 percent, at least, of school children are incapacitated for study because of defective or diseased teeth, and that one-third of all pupils have more or less tooth diseases, and this condition could be practically corrected by systematic inspection two or three times each year.

Children of the poor or liable to have bad teeth and toothache, they loose the teeth early and they are more subject to sickness and injuries. They should be taught in the public schools that diseased teeth lead to indigestion, consequently, a craving for liquor, and this leads to vicious habits and crime. They should be taught the value of sound teeth. Children having diseased mouths are a source of infection in the public schools, therefore a menace to the health of all the pupils. Every school teacher can become an inspector and instructor of oral hygeine.

Next to the instruction in public schools, he advocates public dental hospitals and clinics for the treatment of the worthy poor, and cites the numerous cases in England where this is done either by public provision or by private bequests or subscription. The public elemosenary institutions and hospitals should be provided with dental ministrations. Cases of insanity are many times aggravated if not actually caused by dental disease. An educational campaign should be carried on through public prints. The daily and weekly newspapers and especially the monthly magazines, devoted to home life, etc, are valuable mediums. The subject is worthy of discussion and it would seem that some movement should be started to encourage so desirable a condition of public hygiene.

In the January, 1906, *Items of Interest*, Dr. S. Blatner, of Albany, N. Y., tells of a movement that has been inaugurated in his city to care for the teeth of the poor that is succeeding. He says this work cannot be undertaken in one's private office for very obvious reasons. Under the supporting care of the Albany Guild, a dental infirmary has been equipped and opened to the worthy poor for such treatments as can be măde without heavy expense. The work is under the direct supervision of four ethical practitioners of Albany who report for work twice each month. The clinic is well patronized and very satisfactory results have been secured. A similar plan could be organized in any place in connection with a medical college, a free dispensary or any other public charity or benevolent institution, and by dividing the time properly among a sufficient number of ethical dentists, no great burden would be placed on anyone and a great deal of good service could be rendered to the unfortunate poor, who many times suffer from diseased teeth. In cases where expense for material is involved a suitable charge should be made to cover the expense. If the work is done in connection with some organized charitable institution, there is little chance of the previlege being abused by those who could pay for such services, as the nurses or authorities should investigate every case before admitting them to the clinic, just as they investigate all applicants for other free services.

The Rochester, N. Y., Dental Society organized a free clinic for worthy poor February 27, 1905, at an expense of $1,200. During the first year twenty-four dentists served alternately. Now three dentists are attached who keep the clinics one-half day each day of the week.

The following excerpt from a report by Dr. Swift, of New York City, on practice, to the N. Y. State Dental Society last year, and published in the January 1906 *Cosmos*, page 90, will give a good statement of the present views of prophylaxis.

"Your committee considers oral prophylaxis a most vital subject. To establish and maintain the highest possible standard of prophylaxis should be the aim of every conscientious and truly progressive practitioner. Tiresome and tedious to the extreme—as this work undoubtedly is—requiring the most skilful manipulation, the most careful, painstaking, thoroughly conscientious and constant effort one is capable of lavishing upon it—yet in the experience of the writer, this labor has yielded the most positive and gratifying results he has ever accomplished.

"Prophylaxis and its teachings should begin at the earliest possible age, and instruction as to the proper use of the tooth-brush, dentifrices, mouthwashes, etc., should be the first duty of the dentist to his patient, young or old. after the relief of positive suffering.

"The ignorance displayed by intelligent people regarding the proper use of the tooth-brush is astonishing, and the truth of the old adage, 'It is hard to teach an old dog new tricks,' is evidenced by the fact that patients will so often, especially when pre-occupied or in haste, go back to the method of brushing practiced since childhood. Depending, as we do, so much on the co-operation of our patients, our teachings should begin early, and the importance of frequent and proper brushing of the teeth and gums is so great that it should, if needed, be constantly impressed upon every patient.

"What can be accomplished by prophylaxis in the prevention, arrest and cure of pyorrhea has been demonstrated beyond question.

"Pyorrhea alveolaris with all its symptoms is still increasing, however, and in my experience is manifesting itself very much earlier in life than it did twenty years ago; this fact has forced itself upon my notice, fully developed cases appearing in mouths of children.

"I am now treating a case in the mouth of a girl ten years of age. Her father and mother, two older sisters, and an elder brother are under my care and all have typical cases of pyorrhea. The brother I have only seen once; he has been living in the South, remote from any dentist, and his teeth have received no attention for two and a half years. I found that every tooth in his mouth was involved and he had one of the worst cases of pyorrhea I have ever seen in the mouth of a cultured person. Nearly all of his teeth had been beautifully filled with gold and showed early and conscientious care in that respect, but his dentist had never mentioned that he had even a tendency to pyorrhea."

"It is claimed by many that pyorrhea is the result of purely local causes. Should this increased and earlier manifestation be construed as being entirely due to purely local causes? I firmly believe that malnutrition and faulty circulation as the result of functional derangement and the uric acid and gouty diathesis must be reckoned with as predisposing causes. But aside from all questions as to the etiology of pyorrhea, whether purely local or largely influenced by constitutional conditions, surely our positive duty as practitioners stands out as clearly as the noonday sun. We must ever strive to secure the highest possible standard of prophylaxis, both educationally and practically, particularly with the young, and bend every effort to prevent the dissolution of continuity of the pericementum surrounding the cervical margins of the teeth, thus preventing the opening of a field for the activity of the micro-organisms."

No better presentation of the subject of prophylaxis and pyorrhea has appeared anywhere than was given by Drs. Spalding and Burbridge at the Michigan State meeting last June, and which you will find published in the October 1905, DENTAL REGISTER, pages 492 to 532, inclusive.

In the October, 1905, *Dental Review*, page 930, Dr. Robert Good gives a concise statement of his (which is the younger method) of treating pyorrhea alveolaris. It is largely a surgical operation of scaling the teeth thoroughly under local anesthesia, and a single disinfectant application after scaling of warm chemically pure lactic acid, and then absolute rest.

In the September, 1905, *Review*, page 773, Dr. L. L. Doies raises the question of the infectious character of pyorrhea, and from the date he cites he makes a strong case in favor of infection. We are inclined to think that infection has more to do with the propagation of this trouble than heredity. From careful reading of reports of cases, and from personal observation in families, and also from results of treatment, where all members of the family have been treated we are convinced that there are strong arguments in favor of its propagation as an infectious disease.

Dr. L. P. Bethel, in the *Dental Cosmos* of March, 1905, page 373, has an interesting article on "The Results of Oral Prophylaxis and Care of the Teeth of the Employes of Match Factories." which is interesting in that it shows that the former fate of workers in that portion of match factories where phosphorous is used in a volatile form, is no longer so injurious as to cause extension necrosives of the dental structures. It has been demonstrated that by careful inspection of the teeth of these employes by an expert dentist at frequent intervals and the most rigid attention to filling and polishing the teeth has resulted in avoiding phosphorous poisoning altogether. He also shows from experience in professional prophylaxis that many serious cases of pyorrhea have been cured, and that cases of irritated gums sensitive teeth, etc., have yielded to this treatment with the most satisfactory results. He testifies that patients appreciate the work and most cheerfully compensate for time and skill devoted to it. If it is conscientiously practiced much less decay of the teeth will occur and the frequent loss of teeth from disease of the gums will be greatly hindered. He urges every dentist to do this work and suggests that the public should be informed of its value through some suitable propaganda.

